# Supplementary material for: Salient experiences are represented by unique transcriptional signatures in the mouse brain
Source: eLife. 2018 Feb 7;7:e31220. doi: 10.7554/eLife.31220 (PMC5862526; doi:10.7554/eLife.31220)
Supplement: Transparent reporting form [file elife-31220-transrepform.pdf]

eLIFE
1st Floor
24 Hills Road
Cambridge CB2 1JP, UK
P 01223 855340
W elifesciences.org
T @elife

eLife's transparent reporting form

We encourage authors to provide detailed information *within their submission* to facilitate the interpretation and replication of experiments. Authors can upload supporting documentation to indicate the use of appropriate reporting guidelines for health-related research (see EQUATOR Network), life science research (see the BioSharing Information Resource), or the ARRIVE guidelines for reporting work involving animal research. Where applicable, authors should refer to any relevant reporting standards documents in this form.

If you have any questions, please consult our Journal Policies and/or contact us: editorial@elifesciences.org.

Sample-size estimation

- You should state whether an appropriate sample size was computed when the study was being designed
- You should state the statistical method of sample size computation and any required assumptions
- If no explicit power analysis was used, you should describe how you decided what sample (replicate) size (number) to use

Please outline where this information can be found within the submission (e.g., sections or figure legends), or explain why this information doesn't apply to your submission:

Sample sizes were determined based on our previous experience in these experiments and readouts, as well as 'n' numbers generally found in the literature.

Replicates

- You should report how often each experiment was performed
- You should include a definition of biological versus technical replication
- The data obtained should be provided and sufficient information should be provided to indicate the number of independent biological and/or technical replicates
- If you encountered any outliers, you should describe how these were handled
- Criteria for exclusion/inclusion of data should be clearly stated
- High-throughput sequence data should be uploaded before submission, with a private link for reviewers provided (these are available from both GEO and ArrayExpress)

Please outline where this information can be found within the submission (e.g., sections or figure legends), or explain why this information doesn't apply to your submission:

eLife Sciences Publications, Ltd is a limited liability non-profit non-stock corporation incorporated in the State of Delaware, USA, with company number 5030732, and is registered in the UK with company number FC030576 and branch number BR015634 at the address 1st Floor, 24 Hills Road, Cambridge CB2 1JP | August 2014

All the required information is provided in the method and results sections of the manuscript.
Each experiment was conducted at least twice by different experimenters in the team, with similar results.
Sample sizes are mentioned in legends as ranges for simplicity. Precise sample sizes per condition can be found in Supplementary File_Animal Numbers, as well as in Supplementary File_Raw data and Figure 2 - Figure Supplement 1.
Within each of the experiment performed, a minimum of 3-4 animals were included in each group, most groups include at least 8 animals.
Reported sample sizes refer to number of animals (biological replicates) and are averages of a minimum of 2 technical replicates.

Data exclusion:
No data was excluded except for known technical errors such as inaccurately dissected area, degradation of sample (low RNA yield), and contamination of samples during RNA extraction or qPCR analysis. In some cases, data for a specific gene is missing due to technical errors in loading during qPCR analysis

Statistical reporting

- Statistical analysis methods should be described and justified
- Raw data should be presented in figures whenever informative to do so (typically when N per group is less than 10)
- For each experiment, you should identify the statistical tests used, exact values of N, definitions of center, methods of multiple test correction, and dispersion and precision measures (e.g., mean, median, SD, SEM, confidence intervals; and, for the major substantive results, a measure of effect size (e.g., Pearson's r, Cohen's d)
- Report exact p-values wherever possible alongside the summary statistics and 95% confidence intervals. These should be reported for all key questions and not only when the p-value is less than 0.05.

Please outline where this information can be found within the submission (e.g., sections or figure legends), or explain why this information doesn't apply to your submission:

All the information is provided in the methods and results sections of the manuscript. Statistical analysis was performed with Prism 6.0 (GraphPad, San Diego, CA). Data is presented as Mean and SEM, as described in figure legends. Information pertaining to the appropriate statistical tests is provided in figure legends, wherever applicable. Statistics are reported in Supplementary File 3_Statistics, while the raw data is found in Supplementary File 2_Raw data.

(For large datasets, or papers with a very large number of statistical tests, you may upload a single table file with tests, Ns, etc., with reference to sections in the manuscript.)

Group allocation

- Indicate how samples were allocated into experimental groups (in the case of clinical studies, please specify allocation to treatment method); if randomization was used, please also state if restricted randomization was applied
- Indicate if masking was used during group allocation, data collection and/or data analysis

Please outline where this information can be found within the submission (e.g., sections or figure legends), or explain why this information doesn't apply to your submission:

All animals were assigned to groups pseudorandomly using Latin square design. Moreover, researchers were blinded during collection and analysis of samples. Data was coded and identifiers were unknown to researcher.

Additional data files ("source data")

- We encourage you to upload relevant additional data files, such as numerical data that are represented as a graph in a figure, or as a summary table
- Where provided, these should be in the most useful format, and they can be uploaded as "Source data" files linked to a main figure or table
- Include model definition files including the full list of parameters used
- Include code used for data analysis (e.g., R, MatLab)
- Avoid stating that data files are "available upon request"

Please indicate the figures or tables for which source data files have been provided:

Source data file (“Supplementary File 2_Raw data”; excel file & “Supplementary File 3_Statistics”; excel file are attached) is attached for Figure 1-Figure Supplement 2 and 3, Figure 2, Figure 2-Figure Supplement 1, 3, 4, 5 and 6.
Matlab codes for data analysis are also uploaded.
